# Effects of adult growth hormone deficiency and replacement therapy on the cardiometabolic risk profile

**DOI:** 10.1007/s11102-022-01207-1

**Published:** 2022-02-01

**Authors:** Balázs Ratku, Veronika Sebestyén, Annamária Erdei, Endre V. Nagy, Zoltán Szabó, Sándor Somodi

**Affiliations:** 1 Department of Emergency Medicine, Faculty of Medicine, University of Debrecen, Egyetem tér 1, Debrecen, 4032 Hungary; 2 Doctoral School of Health Sciences, University of Debrecen, Debrecen, Hungary; 3 Department of Emergency and Oxyology, Faculty of Health, University of Debrecen, Debrecen, Hungary; 4 Division of Endocrinology, Department of Internal Medicine, Faculty of Medicine, University of Debrecen, Debrecen, Hungary

**Keywords:** Growth hormone deficiency, Cardiometabolic risk, Growth hormone replacement therapy, Premature atherosclerosis

## Abstract

Adult growth hormone deficiency (AGHD) is considered a rare endocrine disorder involving patients with childhood-onset and adult-onset growth hormone deficiency (AoGHD) and characterized by adverse cardiometabolic risk profile. Besides traditional cardiovascular risk factors, endothelial dysfunction, low-grade inflammation, impaired adipokine profile, oxidative stress and hypovitaminosis D may also contribute to the development of premature atherosclerosis and higher cardiovascular risk in patients with AGHD. Growth hormone replacement has been proved to exert beneficial effects on several cardiovascular risk factors, but it is also apparent that hormone substitution in itself does not eliminate all cardiometabolic abnormalities associated with the disease. Novel biomarkers and diagnostic techniques discussed in this review may help to evaluate individual cardiovascular risk and identify patients with adverse cardiometabolic risk profile. In the absence of disease-specific guidelines detailing how to assess the cardiovascular status of these patients, we generally recommend close follow-up of the cardiovascular status as well as low threshold for a more detailed evaluation.

## Introduction

Adult growth hormone deficiency (AGHD) is a distinct clinical syndrome affecting 200–300 people per million of the population [1]. The disease is characterized by reduced quality of life (QoL) and physical performance, osteoporosis and a coexistence of several cardiovascular risk factors including visceral obesity, adverse lipid profile, endothelial dysfunction and insulin resistance which together with other factors contribute to the higher rates of vascular events as well as excess mortality in hypopituitarism [2–5]. Extensive research of the past decades has shown that AGHD is associated with a low-grade inflammation, oxidative damage, thrombotic tendency, impaired adipokine profile, subclinical left ventricular (LV) dysfunction and high prevalence of metabolic syndrome (MetS) [6–11]. Growth hormone replacement therapy (GHRT) is now considered a safe treatment with beneficial effects on several cardiovascular risk factors, regardless of the underlying cause of GHD [12, 13]. Even though the impact of growth hormone therapy on the cardiovascular mortality of hypopituitarism is still far from being fully elucidated, complex hormone replacement including GH substitution is suggested to result in a mortality that is close to normal [4]. Despite GHRT has been available for more than two decades, disparities regarding the management of AGHD are still substantial [1] indicating that raising the awareness towards the deleterious consequences of the disease as well as the effects of replacement therapy may benefit the patients and may also benefit healthcare professionals.

The aim of this review is to deepen the understanding of the cardiovascular aspects of AGHD, to evaluate the effects of growth hormone replacement on the conventional and novel cardiovascular risk factors, to summarize possible modalities of cardiovascular assessment and to encourage further research in the field.

## Etiology of AGHD

Reduced growth hormone secretion in adulthood can result from pituitary and peri-pituitary tumors, impairment of hypothalamic-pituitary neuroendocrine pathways or local circulatory disturbance associated with surgery, radiotherapy or traumatic brain injury (TBI) [14, 15]. Based on the large international surveillance databases, pituitary adenomas and craniopharyngiomas account for more than half of the cases of AGHD, while idiopathic GHD, Sheehan’s syndrome and TBI are listed as less frequent causes [16]. Although pituitary tumors and their treatment have been consistently reported as frequent causes of AGHD, in the past decade an increasing number of studies revealed that certain non-classical causes of AGHD such as aneurysmal subarachnoid hemorrhage, infections of the central nervous system, sports-related repetitive head trauma and ventricular arrhythmias might be more frequent than previously thought [17]. Childhood-onset GHD (CoGHD) represents approximately 20% of all AGHD cases [18, 19]. GHD in childhood can be classified as idiopathic and organic GHD, with the latter involving congenital and acquired cases [20]. While hormone secretion in patients with idiopathic deficiency often recovers, patients with organic deficiency commonly remain GH deficient in adulthood [19, 20]. Importantly, childhood cancer survivors, especially those who received irradiation, are at greater risk for GHD, which may develop years or even decades after exposure to radiotherapy [14, 21]. The main causes of AGHD are listed in Table 1.

**Table 1:** Main causes of AGHD [14, 17, 19, 20, 22]

Causes of AGHD
**Adult-onset growth hormone deficiency (AoGHD)**
Pituitary adenomas and their treatment
Peri-pituitary tumors: *craniopharyngioma, meningioma, glioma, metastasis*
Irradiation: *cranial, craniospinal or total body irradiation*
Sheehan’s syndrome
Traumatic brain injuries
Vascular catastrophes: *aneurysmal subarachnoid hemorrhage, surgery, stroke*
Central nervous system infections: *bacterial/viral/fungal meningitis*
Infiltrative diseases: *sarcoidosis, tuberculosis, histiocytosis*
Immunologic disorders: *lymphocytic hypophysitis*
Empty sella syndrome
Idiopathic
**Childhood-onset growth hormone deficiency (CoGHD)**
Congenital defects: *anatomical anomalies*, *genetic disorders*
Acquired disorders: *tumors*, *surgery*, *irradiation*, *infiltrative diseases*, *brain injury*
Idiopathic

## Cardiovascular system in AGHD

The GH/IGF-1 axis has an important role in regulating the vascular tone and maintaining the normal endothelial function [23]. Insulin-like growth factor 1 (IGF-1) and GH decrease peripheral resistance through peripheral and central mechanisms [23]. The peripheral actions can be subdivided into endothelial and non-endothelial mechanisms. In endothelial cells, IGF-1 increases the production and release of nitric oxide (NO), which causes smooth muscle relaxation and vasodilation [23, 24]. NO protects the endothelium from atherogenic changes by inhibiting proliferation and migration of smooth muscle cells, reducing LDL-oxidation and inhibiting platelet activation [23–26]. Physiologic NO levels also facilitate vascular repair and angiogenesis [27]. The non-endothelial actions of GH/IGF-1 axis involve stimulating the Na–K-ATPase activity and regulating the expression of the ATP sensitive potassium channel in the vascular smooth muscle [23, 28, 29]. Central regulatory effect via the sympathetic nervous system also contribute to the decrease of peripheral resistance [23, 30]. Moreover, the GH/IGF-1 axis has been suggested to protect the arteries from age-associated changes such as wall thickening and arterial stiffness [31].

**Fig. 1:**
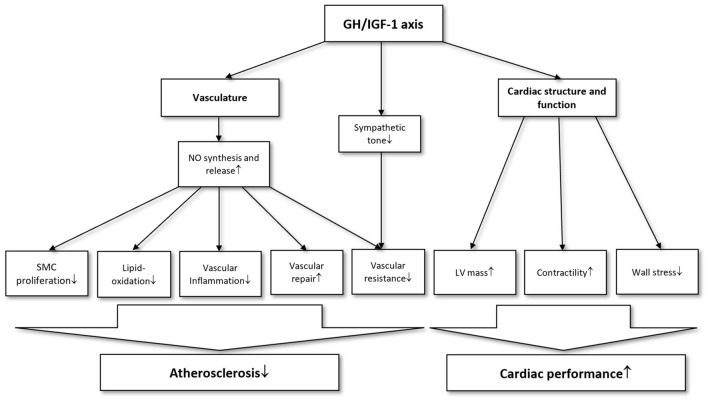
Overview of the cardiovascular effects of GH/IGF-1 axis

In addition to its antiatherogenic and vasculoprotective properties, GH/IGF-1 axis has a considerable effect on the cardiac growth and metabolism [24]. IGF-1 favorably influences the trophic status of the heart by preventing cardiomyocyte apoptosis and promoting hypertrophy of cardiac muscle [24, 25, 32]. Concerning functional effects, GH/IGF-1 axis has been demonstrated to reduce cardiac wall stress and increase contractility [33]. Postulated mechanisms underlying increased contractility involve modulation of intracellular Ca^2+^ levels, increased myofilament sensitivity to Ca^2+^ and upregulation of sarcoplasmic reticulum ATPase (SERCA) levels [34–37]. Furthermore, GH reduces the energy demand of the contractile process through promoting a shift towards V3 myosin isoform, which has a lower ATP-ase activity [23]. The most important physiological effects of GH/IGF-1 axis on the cardiovascular system is summarized in Fig. 1.

### Myocardial structure and function in AGHD

In the past three decades several clinical studies have demonstrated the role of GH in the maintenance of physiological cardiac performance. In an early study, Cittadini et al. found impaired systolic function in untreated adults with CoGHD and detected considerable improvement in cardiac index after 6 months of GHRT [38]. Longobardi et al. reported similar abnormalities in LV function. Based on their findings, patients with AoGHD and CoGHD are not affected differently [39]. Likewise, Amato et al. found significant impairment of LV function as well as reduction of LV mass in GHD patients. Importantly, echocardiographic findings were completely back to normal in response to 6 months of GHRT but disappeared after 6 months of withdrawal [40]. Besides improved cardiac output and stroke volume (SV), Caidahl et al. demonstrated a decrease in diastolic blood pressure (DBP) with 6 months of GHRT [41]. Thuesen et al. conducted the first study in which the cardiovascular function was monitored for a relatively long period of time (38 months). Findings of this study suggested that long-term GHRT results in a normalization of cardiac structure, but the cardiac index and heart rate increased to supernormal levels [42]. Valcavi et al. demonstrated a clear increase in LV mass as well as reversal of diastolic abnormalities after 6 and 12 months of GHRT. All favorable changes disappeared with a 3-month withdrawal, but the increase in LV mass was still detectable [43]. In contrast, another study did not demonstrate LV hypertrophy even after 3–5 years of GHRT [44]. In 2003, a meta-analysis (n = 468) reported positive effect of GHRT on LV mass, LV end-diastolic diameter (LVEDD), intraventricular septum (IVS) thickness and SV; however, hormone substitution was not found to have a significant effect on systolic parameters [45].

These so-called historical studies frequently demonstrated inconsistent findings, which at least partially stem from the heterogeneity of study models and the differences in the administered GH doses, duration of replacement therapy and age of the enrolled patients [46, 47]. Additionally, conventional echocardiography, employed in the majority of these trials, is not considered sensitive enough to detect subtle impairment of LV function [47]. A few studies conducted in the past decade employed cardiac magnetic resonance imaging (CMRI) to evaluate the effect of GHRT. Andreassen et al. conducted the first study employing CMRI and found significantly smaller LV end-systolic and end-diastolic volumes and a trend towards reduced SV in untreated patients compared to healthy subjects; significant association between baseline LV mass index and IGF-1 was also detected. 1-year GHRT resulted in a trend towards an increase in LV mass index, but no significant changes were detected in the cardiac mass [48]. Another notable study reported reduced cardiac mass in AoGHD patients and a significant increase in LV mass index with 1 year of GHRT. Furthermore, untreated GHD patients were also demonstrated to have reduced aortic area; however, this alteration did not change with 1-year GHRT. Significant changes of the right ventricular parameters were not found either [49]. Gonzalez et al. employed echocardiography, CMRI and cardiopulmonary exercise testing and experienced higher systolic blood pressure (SBP), ejection fraction (EF) and LV mass in AGHD patients with no changes in exercise capacity, systolic function and cardiac structure after 9 months of GHRT [50]. More recently, De Cobelli et al. have demonstrated reduced LV mass with normal resting systolic function as well as subclinical diastolic dysfunction in untreated GHD patients. Late gadolinium enhancement, a useful marker of structural abnormalities, was also evaluated: none of the patients showed any pathological areas within the heart [51].

In order to overcome the limitations of conventional 2DE echocardiography, Mihaila et al. employed two-dimensional speckle-tracking echocardiography (2D STE). Despite the fact that conventional echocardiography revealed normal LV systolic function, 2D STE demonstrated impaired LV longitudinal, circumferential and torsion functions in all patients with GHD [9]. In a small sample of patients with GHD, Boschetti et al. found reduced values of coronary flow reserve (CFR), an early marker of impaired myocardial microcirculation, and detected improvement in response to 1-year GHRT [52]. Finally, the most recent meta-analysis confirmed that GHRT was associated with increased EF and found positive effects on the thickness of the IVS and the LV posterior wall (LVPW) and decreased LV end-diastolic volume (LV EDV) which in conjunction with reduction of NT-BNP levels indicated improved systolic function in AGHD patients [53].

### Vascular changes in AGHD

Published results show some controversy concerning blood pressure in GHD patients, but it seems that older patients have a slightly higher risk of developing hypertension. A large cross-sectional study (n = 926), which mainly consisted of patients with AoGHD, reported higher incidence of hypertension compared to general population (22.1 vs. 14.9%) [54] while studies recruiting young patients demonstrated unchanged or even lower blood pressure [38, 40]. The phenomenon of low blood pressure probably resulting from reduced extracellular water volume and low cardiac output has been observed in patients with CoGHD and has not been experienced in adults [55].

Several studies including two meta-analyses demonstrated beneficial effects of long-term GHRT on DBP [53, 56] and improvements are generally explained by the NO-mediated decrease of peripheral resistance and decreased intimal-medial thickness [41]. Concerning the 24-h blood pressure pattern, one study reported high incidence (37.03%) of non-dippers among GHD patients [55] while another study found no changes in the circadian rhythm of blood pressure and heart rate but observed reduction in SBP and DBP after 12 months of GHRT [57].

GHD is associated with premature atherosclerosis [58]. In the process of atherosclerosis, increased carotid intima-media thickness (CIMT), which has long been reported in GHD patients, is considered one of the earliest morphological changes of atherosclerosis [47, 58, 59]. In a prospective study, 1-year GHRT resulted in a significant decrease in CIMT, and the decrement was detected in 21 out of 22 patients [60]. Restoration of CIMT seems to be a relatively early and sustained beneficial effect of GHRT, and it was already observed after 6 months of GHRT [61] and maintained even after 10 years [62]. Other markers of endothelial dysfunction including reduced aortic distensibility as well as decreased flow-mediated endothelium dependent vasodilation were also reported in GHD [63, 64].

### Lipid profile

Dyslipidemia is considered the most significant contributor of adverse cardiovascular risk in hypopituitary patients [65]. Based on a cross-sectional observational study, lipid abnormalities in GHD include higher total cholesterol (TC), LDL-C and triglyceride levels in both sexes, and reduced HDL and Apo A-1 in women [65]. Concerning the effect of GHRT on lipid levels, two meta-analyses reported significant reduction in LDL-C level, one of them also found reduction in TC whereas neither of them demonstrated an increase in HDL-C [56, 66]. On the other hand, a 2-year observational study reported a decrease in LDL-C and TC levels after 6-month GHRT while an increase in HDL-C became significant only after 18 months of GHRT indicating that HDL-C levels may take more time to increase [67]. The latter is also supported by a long-term (15 years) prospective study which demonstrated significant sustained increment of HDL-C levels [68]. As for the triglyceride levels, a 5-year prospective study observed some reduction after years of GHRT, while other studies and a meta-analysis did not [56, 68, 69]. With regard to the degree of LDL-C and TC reduction, 15-year GHRT reduced the TC and LDL-C levels by 1.3 mmol/l (20%) and 1.6 mmol/l (32%), respectively [70].

Although LDL has long been identified as the most atherogenic type of lipoprotein, there is a growing evidence that in addition to the quantity of lipoproteins their quality is also associated with the risk and progression of coronary artery disease [71, 72]. Accordingly, the LDL size appears to be a considerable predictor of cardiovascular events with a predominance of small dense LDL (sdLDL) considered a novel cardiovascular risk factor [72]. Unfortunately, studies of LDL subfractions in GHD are still scarce and demonstrated inconsistent findings. An early study conducted on 30 patients demonstrated that GH-unreplaced patients had an excess of sdLDL compared to healthy controls [73]. Rizzo et al. conducted a small study (n = 14) and concluded that increased sdLDL might be common in GHD and that 4 months of GHRT did not affect LDL size [72]. On the contrary, Salmon et al. observed no significant difference in LDL subclasses between GHD patients and controls [74].

It should be emphasized that appropriate GH replacement is crucial in the management of dyslipidemia in AGHD. The cholesterol lowering effect of GHRT is proved to be at least additive to that of statins [75].

### Inflammatory markers

Today C-reactive protein (CRP) is considered not only an inflammatory biomarker but also a useful prognostic tool in the evaluation of cardiovascular risk [76, 77].

CRP levels in GHD show approximately a fourfold to fivefold increase in both lean and obese individuals, which indicates the presence of a proinflammatory state [78]. In a randomized placebo-controlled study conducted by Sesmilo et al, the mean initial CRP level of the GHD study sample was similar to those observed in patients with a threefold increased risk for future myocardial infarction and a twofold risk for ischemic stroke in the Physicians’ Health Study [7, 76, 79]. In response to GHRT, the reduction in CRP levels was similar to those reported for pravastatin in the Cholesterol and Recurrent Events Study (CARE) [7, 79]. Importantly, the decrease of CRP levels cannot be fully attributed to weight loss or reduction of fat mass, because CRP reduction was also observed without any significant changes in body composition [6].

Some cytokines like tumor necrosis factor alpha (TNF-α) and interleukin-6 (IL-6) are also suggested to take part in the development of endothelial dysfunction and arteriosclerosis in GHD [3]. Although studies on this topic often produced ambiguous results, several studies found negative correlation between GH and proinflammatory cytokines [80]. One study reported significantly elevated TNF-α levels in untreated adolescents with GHD compared to healthy controls. Another study also found higher baseline IL-6 levels in AGHD patients than in controls and demonstrated a significant decrease after 3-month GHRT, suggesting that GH might have an inhibitory effect on these cytokines [81]. Two studies recruiting children with GHD reported similarly elevated levels of TNF-α and reduction in response to GHRT [82, 83].

### Pregnancy-associated plasma protein-A (PAPP-A)

Pregnancy-associated plasma protein-A (PAPP-A) is a high-molecular-weight metalloproteinase that can enhance local IGF-I bioavailability and possibly can alter a number of pathophysiological processes of atherogenesis [84]. Elevated PAPP-A levels were found in acute coronary syndrome and ischemic stroke, moreover PAPP-A is considered a marker for unstable atherogenic plaque [47, 85]. The relationship between PAPP-A levels and atherosclerosis is supported by preclinical studies where the loss of PAPP-A was found to promote plaque regression in mice [86]. A few studies investigating PAPP-A levels in AGHD detected elevated baseline PAPP-A levels which showed significant decrease after GHRT [85, 87].

### Markers of endothelial dysfunction and thrombogenesis

Asymmetric dimethylarginine (ADMA) is an endogenous NO synthase inhibitor and is likely to participate in the development of endothelial dysfunction [88]. Supporting this theory, a meta-analysis (n = 19,842) revealed that patients in the top third of basal ADMA concentrations were at about 40% higher risk of cardiovascular disease compared to those in the bottom third [88]. Krzyzanowska et al. found elevated ADMA levels in hypopituitary patients, but regarding their ADMA levels no difference was found between GH deficient and GH sufficient patients [89]. Another study conducted on 31 adult GHD patients reported decreased ADMA levels after 6-month GHRT [90]. Interestingly, a significant decrease in ADMA levels were also reported in healthy subjects after 10 days of GH therapy [91].

Additionally, GHD is associated with alterations of coagulation and fibrinolysis [92]. Elevated levels of plasminogen activator inhibitor-1 (PAI-1) [92–94], fibrinogen [92, 94] and von Willebrand factor (vWF) [93] and a decrease in PAI-1 [94] in response to GHRT were reported in a relatively small number of studies. Higher E-Selectin [93, 95], P-selectin [96] and ICAM-1 [93] levels were also demonstrated in patients with GHD, and significant reduction in these adhesion molecules after 12 months of GHRT was also reported [96]. Furthermore, Cakir et al. observed a higher rate of Protein S deficiency in AGHD compared to general population and normalization with substitution [97]. In a small study (n = 21) Miljic et al. found a significant increase in prothrombin time (PT) and activated partial thromboplastin time (aPTT) after 12 months of GHRT [98].

**Fig. 2:**
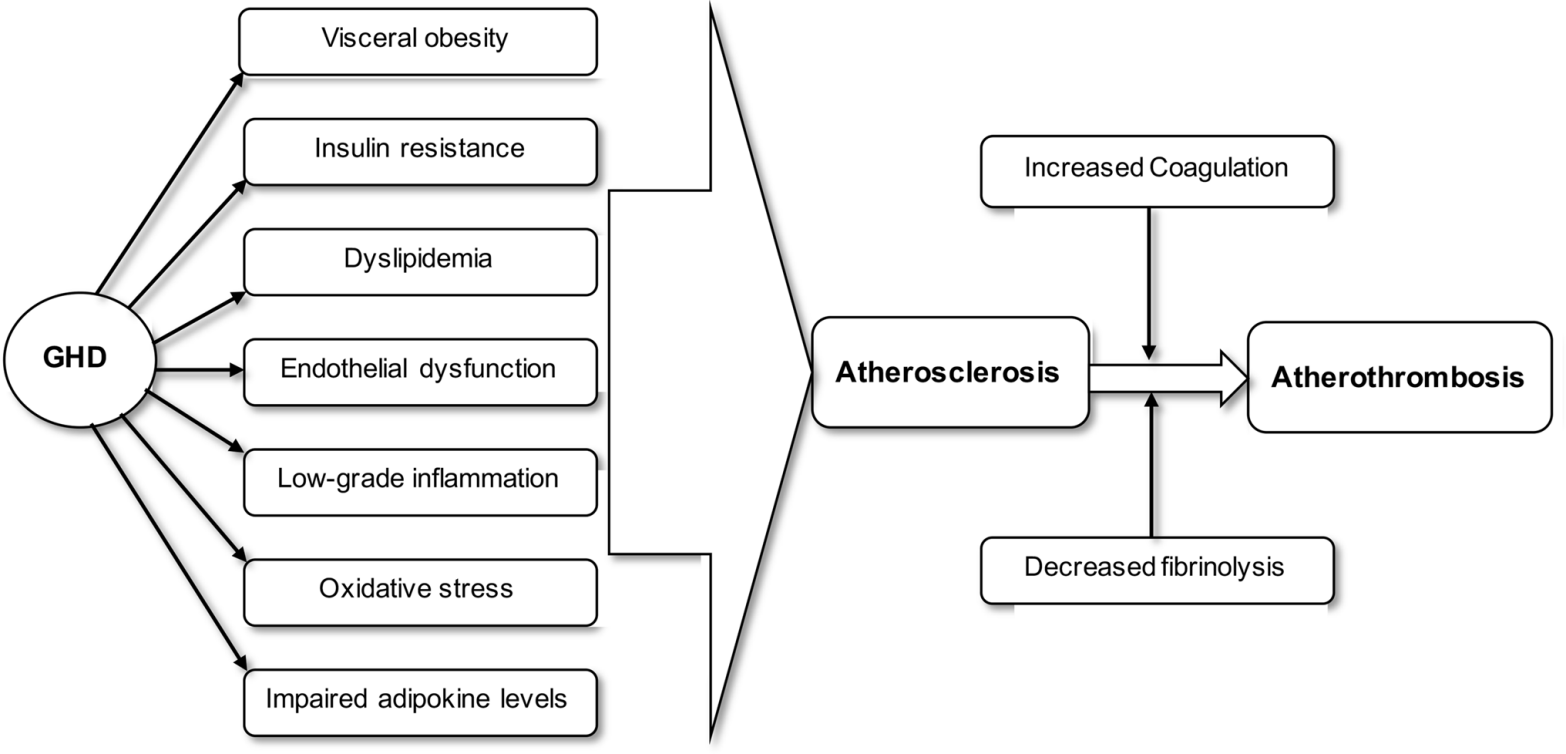
Unfavorable effects of GHD may lead to vascular consequences

Although, data obtained from these studies show some inconsistency, it is reasonable to state that GHD is associated with a mild prothrombotic state which may act as an additional contributor to atherothrombotic events (Fig. 2).

### Oxidative stress

Based on clinical and preclinical studies, oxidative stress (OS), defined by an imbalance between radical species and antioxidant defense, may have an important role in the development of metabolic alterations and atherosclerosis in GHD [99]. Accordingly, in an early study Evans et al. detected increased levels of lipid-derived free radicals and experienced improvement with 3-month GHRT [100]. On the contrary, another early study concluded that OS is not an important factor in the proatherogenic state in GHD [101]. More recently, Mancini et al. evaluated new parameters of OS as well as parameters of macromolecular oxidative damage and have found increased Total Antioxidant Capacity levels (as a consequence of greater antioxidant production to balance OS) in GHD reinforcing that OS is increased in patients with AGHD [8].

### Adipokines

Besides regulating fat distribution, GH also modifies the endocrine function of the adipose tissue [102]. Adipokines participate in the regulation of energy balance, lipid and carbohydrate metabolism and inflammatory processes [102, 103]. It is also suggested that the GH/IGF-1 axis can influence the secretion of adipokines, which, in turn, can mediate metabolic effects of the GH/IGF-1 axis [102, 103]. The most frequently studied adipokines are leptin and adiponectin [104]. Leptin, the first adipokine to be discovered, shows a positive correlation with fat mass and is likely associated with OS and vascular damage [3, 105]. In GHD, some but not all studies reported higher levels of leptin [85, 106] and a decrease in response to GHRT [106, 107]. Adiponectin, the most abundant adipokine, is known for its antiatherogenic, insulin sensitizing and anti-inflammatory properties and has a key role in obesity-related diseases [108, 109]. Fukuda et al demonstrated significantly lower adiponectin levels in GHD when compared to acromegalic patients, but no differences were found compared with adiponectin levels of normal subjects [108]. Concerning the effect of GH substitution, elevated [110] and unchanged [111] adiponectin levels were also demonstrated in different trials. More recently, Wang et al. reported increased adipsin levels in GHD patients compared to controls, and found a significant correlation with cardiometabolic risk factors [10]. Adipsin itself is found to improve and maintain pancreatic β-cell function and to increase insulin secretion to glucose [112]. In obesity, higher adipsin production is considered a form of compensatory mechanism for normalizing lipid and glucose metabolism [113]. This hypothesis may also explain the increased levels found in patients with AGHD.

### Vitamin D status

Hypovitaminosis D represents an environmental risk factor for cardiovascular diseases and is associated with hypertension, dyslipidemia, endothelial dysfunction and impaired insulin metabolism [114, 115]. Findings from the past decade suggest a functional relationship between the GH/IGF-1 axis and vitamin D, which then leads to the assumption that hypovitaminosis D may contribute to the higher cardiovascular risk profile in GHD [115]. One study conducted in 41 GH-naive patients revealed a higher prevalence of vitamin D deficiency compared to controls (51% vs.14.6%). The presence of hypovitaminosis D was also found the most powerful predictor of the prevalence of dyslipidemia and hypertension in GHD [115]. Another study with a larger sample (n = 129) found an even higher prevalence of hypovitaminosis (71.9%) in AGHD [116]. Interestingly, a subgroup of GH deficient patients fulfilling the criteria for MetS had a significantly lower mean 25(OH)D concentrations [116], which may indicate that lower mean 25(OH)D concentrations are associated with higher cardiovascular risk in patients with AGHD. Although favorable effects of vitamin D supplementation on the cardiovascular risk are not proven, vitamin D testing may help to identify patients with high adverse cardiovascular risk in AGHD [116].

## Visceral obesity in AGHD

GH has important direct actions on adipocytes resulting in increased lipolysis and decreased lipogenesis. Moreover, GH also produces indirect actions influencing adipocyte growth and differentiation [117]. Obesity, which is a common finding in AGHD, is considered a consequence of blunted lipolysis leading to accumulation of mainly visceral fat [118].

Several mechanisms have been proposed to explain the link between visceral obesity and GHD. First, GHD is associated with a decreased hormone-sensitive lipase (HSL) activity, which has an essential role in the process of lipolysis [118]. Second, GH may influence the expression of CIDE-A (cell-death-inducing DFF45-like effector) protein which has been associated with lipid accumulation and inhibition of lipolysis [118]. Third, GH regulates tissue glucocorticoid exposure by inhibiting the 11β-hydroxysteroid dehydrogenase type one (11β-HSD1) enzyme that interconverts inactive cortisone to active cortisol [119]. The inhibitory effect of GH on 11β-HSD1 leads to less cortisol production in the visceral adipose tissue, while GHD causes enhanced enzyme activity and visceral adiposity through local cortisol excess [120, 121].

According to early studies, fat mass is increased by 7% in GHD while lean body mass (LBM) decreased by 7–8% compared with predicted values [122]. Beneficial changes of GHRT on body composition are demonstrated in several studies [56, 123, 124]. One meta-analysis reported an increase in LBM of 2.7 kg and a reduction in fat mass of 3.1 kg in response to substitution [56]. Conversely, changes in body mass index and total body weight are observed in some but not all studies, and it seems that they do not necessarily change during GHRT [56, 123].

The effects of GHRT on body composition are considered dose-dependent with lower doses resulting in somewhat weaker effect on fat mass [56]. Different GH-responsiveness in men and women is also documented and is explained by the effect of sex hormones. Testosterone has an enhancing effect on IGF-1 production while oral estrogen administration decreases hepatic IGF-1 synthesis; therefore, similar doses of GH cause less IGF-1 production and not so pronounced fat loss in women [124, 125]. The unfavorable effect of oral estrogen can be eliminated by administering transdermal or transvaginal estrogen instead. However, in a recent survey, only 10% of the endocrinologists considered switching from oral estrogen to a transdermal or transvaginal route [1].

Findings obtained from an RCT suggest that discontinuation may diminish the beneficial effects of long-term GHRT, and patients with a 4-month period of placebo treatment increased their waist circumference by 1.4 cm [126] which increases the cardiovascular risk by at least 2% [126, 127].

As for the duration of the improvements, in a 15-year observational study, lean soft tissue showed a sustained increase, and although, body fat returned to the baseline level, it was still well below the baseline when expressed as a percentage of total body weight [68].

## Insulin resistance and diabetes in treated and untreated patients with AGHD

Insulin resistance and type 2 diabetes mellitus (T2DM) are well-established cardiovascular risk factors. Based on epidemiological evidence, a higher plasma glucose level not necessarily reaching the diabetic or even impaired glucose tolerance threshold also increases the cardiovascular risk [128].

Untreated patients with AGHD are found to be insulin resistant, which shows strong association with older age and percentage of fat mass [129]. Since GHRT leads to favorable changes of body composition, GH substitution would be expected to improve insulin sensitivity [130]. Conversely, studies in this field often reported unchanged or even deteriorating insulin sensitivity in response to GHRT [130]. Recently a meta-analysis demonstrated that short-term GHRT (6–12 months) resulted in deterioration of glucose metabolism including fasting insulin (FI), fasting plasma glucose (FPG), glycated hemoglobin (HgbA1C) and homeostasis model of assessment-insulin resistance (HOMA-IR), however, apart from FPG, negative effects of GHRT on these parameters are not observed in longer (> 12 months) GHRT [131].

Based on an international database which follows 6050 patients, the prevalence of diabetes mellitus is increased (SPR: 1.13) in untreated GHD, which is largely explained by the impaired body composition [132]. As far as treated GHD is concerned, another international follow up study found no evidence of increased prevalence of diabetes among patients receiving GHRT and concluded that higher susceptibility to diabetes is largely related to the increased prevalence of obesity and MetS in GHD rather than the GHRT itself [133].

In general, lower initial GH doses are recommended when GHD develops in a diabetic patient and antidiabetic therapy may need adjustment if GH replacement is started [22]. However, at present there are no specific recommendations regarding antidiabetic medications in AGHD. Interestingly, the dipeptidyl peptidase-4 inhibitor sitagliptin has been recently demonstrated to enhance endogenous GH and IGF-1 secretion in women [134] which may be beneficial in patients with coexisting diabetes and AGHD and is worth being examined in future research.

## Metabolic syndrome in patients with AGHD

The prevalence of MetS is about 20–30% in the general population while it was found considerably higher in AGHD affecting about half of the patients [11, 135, 136]. In an analysis of 2479 patients with severe AGHD, the prevalence of MetS was 43.1% and 49.1% according to the definitions by NCEP-ATPIII and IDF respectively [11]. When comparing to patients with no MetS, patients with untreated AGHD and MetS also demonstrated higher prevalence rate for coronary and cerebrovascular morbidity [11].

It can be stated that GH substitution itself does not reduce the prevalence of Mets in patients with AGHD. In a recent analysis (n = 1449), 1-year GHRT did result in beneficial changes including a decrease in the percentage of patients with abnormal waist circumference, but the prevalence of MetS did not change [136]. Interestingly, patients without MetS proved to be much better GH responders and showed more favorable changes in the components of MetS than patients with MetS [136]. Another study demonstrated ongoing beneficial effects on lipid profile, but the prevalence of MetS increased significantly (57.1% vs. 32.7%) after 10 years of substitution and the increase was found higher than anticipated as a consequence of aging [70].

## Psychological well-being and cardiovascular health

Impaired QoL, measured by the disease specific QoL-AGHDA and a series of other questionnaires, is a well-documented feature of AGHD [122, 137]. Memory, concentration, energy and vitality are affected most seriously in untreated hypopituitary adults, but depressed mood, social isolation and anxiety are also frequently reported [137, 138]. Timely and long-term GHRT is proved to be capable of improving QoL with older patients reaching the general population levels in 2 years [137].

From the clinical viewpoint, the positive change in the QoL is an important marker of the efficacy of hormone substitution, but it is generally not considered to be a possible contributor to cardiovascular health. As a matter of fact, negative psychological factors including anxiety and depression have a well-established role in the development and progression of cardiovascular diseases [139]. Anxiety disorder, which is found more frequent in GHD than in general population (38% vs. 13%) [140] is associated with an increased risk of cardiovascular mortality as well as specific cardiovascular diseases such as coronary heart disease, stroke and heart failure [141]. Social isolation, which normalizes early in response to GHRT [137], is associated with a 1.5-fold increased risk of coronary heart disease in adults and proved to worsen the prognosis when coexists with chronic conditions [142]. On the other hand, a growing body of evidence shows that positive psychological factors can affect cardiovascular health beneficially through healthier biological and behavioral processes such as healthier stress responses, better lifestyle choices and better compliance and adherence to medications [141].

In synthesis, improving QoL and psychological well-being not only improve patients functioning but may also have a favorable effect on the overall cardiovascular status and disease prognosis in AGHD.

## Differences of cardiovascular risk profile in subgroups of AGHD patients

AGHD is considered a heterogenous condition in which the differences in the onset of the deficiency and the etiology not only influence the clinical presentation but also have an impact on the cardiovascular risk profile [2]. The marked differences in baseline characteristics and response to GHRT between CoGHD and AoGHD have long been recognized and documented. Basically, AoGHD is considered a metabolic disorder while CoGHD is a more complex syndrome including developmental as well as metabolic components [2, 143]. Patients with CoGHD are usually shorter, have lower initial BMI, waist-hip ratio (WHR), LBM and serum IGF-1 concentration as well as less disturbed baseline lipid profile including lower LDL-C and higher HDL-C levels [2, 144]. In a comparative, prospective study, initially, reduction in TC levels was observed only in patients with AoGHD, however, there were no differences in lipid concentrations between the two groups after 5 years of GH treatment indicating that lipid abnormalities of both CoGHD and AoGHD can be improved with long-term GHRT [144]. Besides the onset of the deficiency, the influence of other hormone deficiencies and their replacement may also have an impact on the cardiovascular risk [145]. Accordingly, in a surveillance study, patients with multiple pituitary hormone deficiencies (MPHD) presented worse cardiometabolic risk profile than those with isolated GHD [145]. Certain etiologies like craniopharyngioma are also associated with more adverse cardiovascular risk profile [146]. Studies comparing craniopharyngioma patients with patients having non-functioning pituitary adenoma demonstrated that craniopharyngioma patients are more obese and dyslipidemic and less sensitive to the positive effects of GHRT on fat mass and lipid profile which, at least partly explain the higher mortality rate observed in this subgroup [146–148].

## Cardiometabolic risk profile in patients with biochemical GHD

In the past decades, a few clinical conditions were identified to be associated with reduced growth hormone secretion, which do not meet the standard criteria for true GHD. This alteration in GH secretion, often referred to as biochemical or functional GHD, has been reported in a subgroup of patients with human immunodeficiency virus (HIV) infection as well as in patients with chronic heart failure (CHF) and are suggested to have negative impact on disease progression [149–151].

Lipodystrophy is a common endocrine abnormality in HIV-infected patients receiving highly active antiretroviral therapy (HAART) [150]. HIV-related lipodystrophy is characterized by an increase of visceral fat mass which, given the well-known effects of GHD on fat distribution, directed the attention towards pituitary GH secretion as a potential contributor to the development of lipodystrophy [150]. Eventually, biochemical GHD was found to be common in HIV infection, affecting about one-third of the patients [150]. Although, the significance and mechanism of impaired GH secretion in HIV is far from clear, patients with biochemical GHD demonstrate alterations that are frequently observed in true GHD, which supports the presence of a clinically significant GH deficiency [150, 152]. Cardiovascular diseases are leading causes of death in patients with HIV infection [153]. Risk factors in HIV probably involve traditional cardiovascular risk factors as well as factors associated with the antiretroviral therapy [153]; however, biochemical GHD characterized by increased visceral adiposity, dyslipidemia, inflammation and altered glucose metabolism may also contribute to the higher cardiovascular mortality among these patients [152].

CHF is often associated with multiple hormonal deficiencies which identifies a subgroup of patients with worse prognosis and higher mortality [154]. GHD, which affects approximately 30% of these patients, is considered a key component among anabolic hormone deficiencies [151, 155]. The presence of GHD in CHF is associated with worse clinical status, reduced QoL, worse depression scores, LV remodeling, lower physical performance, increased NT-proBNP levels and higher all-cause mortality [151]. Circulating IGF-1 levels in CHF can be lower, unchanged or higher compared with healthy subjects; however, low IGF-1 shows correlation with systolic dysfunction, skeletal muscle performance and neurohormonal and cytokine activation [156, 157]. A number of studies have attempted to evaluate the effects of growth hormone therapy in CHF; unfortunately many of them provided conflicting results probably due to differences in the administered treatment and inclusion of patients regardless of their GH/IGF-1 profile [156]. Cittadini et al. conducted a randomized, single-blinded, controlled trial on 158 patients with CHF (NYHA II-IV) and found significant improvement in exercise capacity, QoL and LV structure and function in patients receiving GH replacement [154]. When they assessed the patients in a 4-year follow-up study, they found that long-term GH replacement in patients with CHF resulted in increased EF, decreased LV end-systolic and end-diastolic volume, reduced wall stress as well as reduced hospitalization rate due to CHF worsening [158].

## Cardiovascular evaluation in AGHD

Adults with GHD have an increased risk of cardiovascular morbidity and mortality; therefore, it is essential for these patients to undergo regular evaluation of cardiovascular parameters. Based on the recent guideline, parameters that are considered to be monitored at 6–12-month intervals include blood pressure and heart rate while more thorough evaluation involving electrocardiography, echocardiography and carotid echo-Doppler examination can be performed if clinically indicated [22]. In the absence of disease-specific guidelines detailing how to assess the cardiovascular 

status of these patients, we generally recommend close follow-up of the cardiovascular status as well as low threshold for a more detailed evaluation.

### Echocardiography

Due to its widespread availability, echocardiography is commonly used for the diagnosis and management of cardiovascular diseases [159]. Echocardiographic findings in AGHD may involve reduced LV mass especially in young patients and impaired LV function [45]. Several studies demonstrated positive effects of GHRT on LV mass [45], IVS [45, 53] and LVPW [45, 53] probably resulting from the hypertrophic effect of GH [76] and contributing to the improved cardiac performance in treated patients. Besides cardiac MRI, echocardiography is considered the best modality to assess LV mass [160] with several methods available for the effective calculation including M-mode echocardiography, 2DE and 3DE [159]. LV mass is generally presented as LV mass index (LVMI) where LV mass is indexed to height, weight or body surface area allowing it to compare different patients with different stature [159]. Based on the recommendations of the American Society of Echocardiography (ASE), 2DE is considered an advisable method for follow-up of changes of LVMI in an individual patient [159]. Ejection fraction (EF) is the most widely-used and accepted echocardiographic parameter of the LV systolic function [161]. In AGHD a substantial number of studies employing echocardiography and the highly sensitive radionuclide angiography found reduced EF, which improved with GHRT [46, 53, 162]. At present, the biplane method of disks (modified Simpson’s rule) is the recommended 2 DE method to assess LVEF with EFs lower than 52% for men and 54% for women considered indicative of LV systolic dysfunction [159, 161].

Because of the growing need for more reliable measures of systolic function, new techniques including 3D and STE are expected to become widely available in the near future [161]. STE allows angle-independent evaluation of myocardial function and it also has a potential to detect subclinical systolic dysfunction not evident by LVEF assessment [163, 164]. To the best of our knowledge, there was only one study in which both conventional 2DE and 2D STE were used to evaluate the cardiac function in AGHD. In this study (n = 52) more than half of the patients had an LVEF within normal ranges when assessed with conventional 2DE [9]. In contrast, 2D STE revealed decreased global longitudinal (GLS) and circumferential (GCS) strain in all patients with GHD [9]. It is widely-known that patients with reduced LVEF pose a high risk of all-cause mortality but this is not applicable to patients with an LVEF higher than 40–45% [161, 165]. Conversely, GLS, the most commonly used strain-based measure of global systolic function, provides prognostic information when EF is normal or near-normal [159, 165]. Accordingly, a meta-analysis involving 5721 patients confirmed that GLS impairment is superior to LVEF in the prediction of death and major cardiac events [165].

### Cardiac magnetic resonance imaging (CMRI)

CMRI is now considered a reference standard for the evaluation of cardiac function and morphology because of its high accuracy and independence of acoustic windows. In the clinical setting, CMRI can be utilized to evaluate several parameters including tissue composition, wall motion, blood flow and metabolism [166]. From another aspects, due to its high reproducibility, CMRI allows a substantial reduction in patient numbers compared to conventional echocardiography [167] making it a reasonable modality in clinical trials with rare diseases like AGHD, which often face difficulties with small sample sizes. Studies employing CMRI in AGHD confirmed the previously reported morphological changes and reported reduced LVMI in untreated patients [49, 51] which increased in response to GHRT [48, 49]. One study also reported reduced aortic area in untreated patient [49]. An evaluation of gadolinium enhancement, a useful tool for tissue characterization, was performed in two studies in AGHD. The basis for the examination is that several minutes after its administration, gadolinium-based contrast medium shows a greater distribution volume in necrotic or fibrotic myocardium than in viable myocardium. This is commonly referred to as late gadolinium enhancement or delayed enhancement [166, 168]. In AGHD, late enhancement was detected only in patients with a known history of ischemic heart disease, making it clear that cardiac morpho-functional alterations in AGHD are not related to fibrosis or inflammatory degeneration [49, 51].

### Carotid ultrasonography

Ultrasound examination of the carotid arteries allows detection and characterization of carotid plaques and measurement of CIMT which are particularly important in the cardiovascular risk assessment [169–171]. Increased CIMT, defined as IMT higher than 0.9 mm, is considered an early sign of atherosclerosis and is associated with increased risk of stroke and myocardial infarction [172]. Plaque is generally defined as a focal thickening that is at least 50% greater than the surrounding sites or as a focal region with CIMT measurement ≥ 1.5 mm that protrudes into the arterial lumen [173, 174]. Based on the current recommendations, examination of both IMT and plaque area is of greater value in the estimation of cardiovascular risk, than examining CIMT alone [174]. In AGHD not all but several studies demonstrated increased CIMT and restoration after hormone substitution [58–60]. In one study, ultrasonic imaging of both the carotid and femoral arteries revealed that GH deficient patients not only had increased CIMT, but the percentage of individual arteries with a plaque was also higher in them than in healthy controls [58]. From the therapeutic viewpoint, it is important to emphasize that arterial plaque burden should be considered as a risk modifier and significant plaque on carotid ultrasonography automatically places the patient into the very high risk category where active management of all risk factors should be warranted [169].

### Electrocardiography

Electrocardiography is a non-invasive and unexpensive test, which is universally incorporated in the routine cardiological diagnostics. It is a safe and easily available modality for cardiovascular risk stratification [175]. However, according to the current guidelines, standard 12-lead electrocardiogram does not have an established role in the cardiovascular risk assessment [176]. In fact, the US Preventive Services Task Force (USPSTF) does not recommend employing resting or exercise ECG to prevent cardiovascular events in asymptomatic adults [176, 177].

GHD is known to influence sympathetic outflow and have an unfavorable effect on the structure and function of the cardiovascular system [23], but a little is known about how GHD and GHRT affect arrhythmogenesis. Only a few studies have tested the hypothesis that higher cardiovascular mortality in GHD might be associated with cardiac autonomic dysregulation. Heart rate variability (HRV) assessment is an accepted non-invasive method for assessing the cardiac autonomic tone [178]. Measurement of HRV can be classified into time-domain variables usually derived from 24-h ECG recordings and frequency-domain variables which are derived from power spectral analysis [178]. Leong et al. performed a small study (n = 14) using frequency-domain parameters and detected decreased sympathetic tone in untreated GH deficient adults [179]. Previous studies linked depressed symphathovagal tone to higher mortality in postmyocardial patients and in patients with congestive heart failure [180]. In another study, Leong et al. also evaluated the effect of 6 months GHRT on HRV and found similar cardiac autonomic tone in treated GHD patients and in healthy controls suggesting that cardiac autonomic dysfunction could be corrected by GH replacement [180]. Studies using time-domain variables also found decreased sympathetic tone in adults with untreated GHD, which increased significantly with GHRT [178, 181]. On the contrary, Boschetti et al. did not detect symphathovagal imbalance in unreplaced patients, although, patients having GHD for more than 2 years were not included in the study [182]. Consequently, it cannot be excluded that patients untreated for more than 2 years may exhibit cardiac autonomic dysfunction [182].

In the past two decades, different electrocardiographic markers including the Tp-e interval (distance between the peak and end points of T-wave) and Tp-e/QT ratio (arrhythmogenic index) have been identified as predictors of ventricular arrhythmias [183, 184]. Tp-e interval and Tp-e/QT ratios have been investigated in many conditions affecting the cardiovascular system including diabetes and hypothyroidism and have been found indicative of higher risk for ventricular arrhythmias [184, 185]. Despite the well-documented cardiovascular involvement, clinical electrocardiographic studies are still scarce in AGHD, and the abovementioned electrocardiographic markers of arrhythmias have not been investigated.

## Conclusions

AGHD is a clinical entity characterized by numerous metabolic abnormalities and increased cardiovascular morbidity and mortality. Adequate GH replacement produces favorable changes in body composition, lipid profile and QoL, affects cardiac structure and function positively and indirectly reduces mortality in hypopituitary patients. Nevertheless, it is also evident that GHRT in itself is not capable of eliminating all cardiometabolic abnormalities observed in AGHD; therefore, active screening and treatment of cardiovascular risk factors should be integrated in the routine management. Greater awareness of the advantages of growth hormone substitution among patients and healthcare professionals would be necessary to improve adherence and outcome in AGHD.

## Data Availability

Not applicable.
